# Potential Role of Red Yeast Rice in the Prevention and Treatment of Alzheimer's Disease

**DOI:** 10.7150/ijms.135847

**Published:** 2026-08-22

**Authors:** Qining Quan, Ronghua Pan, Haolin Liu, Lizhong Zheng, Rui Zhang

**Affiliations:** 1 Zhejiang Chinese Medical University, Hangzhou, Zhejiang 310053; 2 Zhejiang Sanhe Bio-Tech Co., Ltd, Jiangshan, Zhejiang 324100

**Keywords:** Red Yeast Rice, Monascus, Alzheimer's disease, Monascus pigments, monacolin K, Lovastatin

## Abstract

Red Yeast Rice (RYR) fermented from *Monascus* is a traditional fermented product with both dietary and medicinal uses and is widely used for lipid regulation. Clinically, it is utilized to lower blood lipid levels and manage hyperlipidemia. Contemporary research indicates that RYR is abundant in secondary metabolites, such as monacolin K, *Monascus* pigments, and γ-aminobutyric acid, which contribute to its lipid-lowering, blood sugar-reducing, anti-inflammatory, neuroprotective, and antihypertensive properties. These metabolites may play a significant role in the prevention and treatment of Alzheimer's disease (AD) through mechanisms that include cholesterol-dependent pathways, anti-neuroinflammation, antioxidant activity, neuroprotection, modulation of intestinal flora, inhibition of high-risk factors for AD, and suppression of AD-related β-amyloid peptide deposition and tau protein hyperphosphorylation. Consequently, RYR demonstrates potential for a multi-component synergistic approach to preventing and treating AD. However, current research on the use of RYR for AD prevention and treatment remains limited. This review focuses on the primary functional substances in RYR, elucidates the anti-AD effects and mechanisms of each metabolite, analyzes the advantages of its multi-component and multi-target properties, and provides a foundation for the further development of RYR.

## Introduction

With global population aging, the prevalence and mortality of Alzheimer's disease (AD) continue to increase. As of 2023, approximately 50 million people worldwide were affected by AD, and this number is projected to reach 152 million by 2050 1. AD not only seriously harms the personal health of patients, but also brings heavy care and economic burdens to families and society. It is currently estimated that the total social burden of AD patients worldwide exceeds 958 billion US dollars and is expected to increase significantly by 2050 2. Although some drugs for improving AD conditions, such as donepezil, have been developed, these drugs cannot fundamentally reverse the lesions of AD patients, and the toxic and side effects caused by long-term use are obvious. At present, there are still no drugs that can effectively prevent or treat Alzheimer's disease in clinical practice. Therefore, the search and research for multi-target and synergistic natural medicines for the prevention and treatment of AD is a future trend.

Red Yeast Rice (RYR), a traditional Chinese medicine with both medicinal and culinary origins, boasts a history of extensive applications spanning thousands of years. It is rich in various bioactive metabolites that influence lipid metabolism, neuroinflammation, oxidative stress, and the regulation of the gut-microbiome-brain axis. Consequently, this review investigates the potential of bioactive metabolites in RYR in the prevention and treatment of AD. By examining the pathological mechanisms and risk factors associated with AD, this review provides a comprehensive evaluation of the multiple functional components of RYR. Ultimately, we aim to elucidate the anti-AD effects and mechanisms of the secondary metabolites found in RYR, thereby providing a foundation for further research and the development of applications aimed at preventing and treating AD.

RYR is produced by fermenting rice with filamentous fungi of the genus *Monascus*. During fermentation, *Monascus* grows on the rice substrate and synthesizes a variety of bioactive metabolites, which are then accumulated in the fermented product. These RYR-derived metabolites include monacolin K, *Monascus* pigments, γ-aminobutyric acid (GABA), and other fermentation-related compounds. This process is presented in Figure 1. Therefore, in this review, the term “RYR-derived metabolites” refers to bioactive compounds generated during *Monascus*-mediated fermentation and retained in the final RYR product.

## Main functional substances and functions in RYR

RYR is a traditional fermented product that originated in China. It is produced through solid or liquid fermentation using specific *Monascus* species on steamed rice and other grain substrates. RYR has long been used as a food colorant, flavor enhancer, brewing material, and traditional Chinese medicinal product. Ongoing research into RYR components has revealed its potential effects in lowering blood lipids and blood sugar, as well as its anti-inflammatory and antioxidant properties. Concurrently, the optimization of *Monascus* strains and the application of pure fermentation technology, which incorporates rigorously screened and identified superior* Monascus* strain, have led to its increased use in the medical and health sectors. The anti-Alzheimer's disease (AD) potential and multi-target synergistic effects of RYR may be attributable to its abundant secondary metabolites, including monacolin K, *Monascus* pigments, and GABA. These functional compounds provide a foundational basis for the prevention and treatment of AD through the use of RYR.

### Monacolin K

Japanese scientist Endo et al. (1979) first discovered and extracted monacolin K from RYR in 1979 during their search for hypolipidemic substances, subsequently confirming its hypolipidemic activity 3. Alberts et al. (1980) later isolated mevinolin, now widely known as lovastatin, from cultures of *Aspergillus terreus,* and subsequently established that major monacolin K in RYR is the natural form of lovastatin (acid lovastatin) 4. Since that time, monacolin K has been commercially purified and marketed under the trade name lovastatin. Monacolin K shares a similar chemical structure with 3-hydroxy-3-methylglutaryl coenzyme A (HMG-CoA) and competitively inhibits HMG-CoA reductase, the rate-limiting enzyme in the mevalonate pathway of cholesterol biosynthesis 5. By inhibiting HMG-CoA reductase, monacolin K reduces mevalonate formation and downstream cholesterol synthesis, thereby exerting its lipid-lowering effect 5. Commercial lactone lovastatin requires hydrolytic activation to its active acid form in plasma and liver cells. Long-term or high-dose lovastatin use may be associated with elevations in serum alanine aminotransferase (ALT) and aspartate aminotransferase (AST) levels and rare clinically significant liver injury or acute liver failure 6. The acidic lovastatin produced by the natural fermentation process has lower crystallinity, higher oral bioavailability, and better safety in toxicological evaluation 7. As a lipophilic statin and a secondary metabolite of *Monascus*-fermented RYR, monacolin K can cross the blood-brain barrier and has been reported to possess neuroprotective activity in neuronal cell models 8. Cohort evidence suggests that lovastatin use may be associated with slower cognitive decline or potential clinical benefits in patients with AD 9. Together, these experimental and clinical observations indicate that monacolin K/lovastatin may represent a promising RYR-derived candidate for AD-related intervention. However, further experimental and clinical studies are warranted to clarify the AD-related potential of monacolin K in RYR.

### *Monascus* pigments

*Monascus* pigments are another major secondary metabolite in RYR 10. More than 100 types of *Monascus* pigments have been identified so far, and they are divided into six major categories: monascorubramine, rubropunctamine, monascorubrin, rubropunctatin, ankaflavin and monascin 10. *Monascus* pigments are polyketide compounds containing a variety of functional groups. These functional groups contribute to the antioxidant, anti-inflammatory, hypolipidemic, anti-diabetic, and gut microbiota-regulating effects of *Monascus* pigments 11. Lin et al. (2023) demonstrated through *in vivo* experiments in mice and cell-based *in vitro* experiments that the anti-inflammatory and antioxidant effects of *Monascus* pigments can inhibit neuroinflammation induced by Aβ40 12. Their blood pressure and blood sugar-lowering effects may attenuate the induction and promotion of AD by metabolic syndrome 13. Several studies have demonstrated that *Monascus* pigments can modulate intestinal microbiota 14, therefore potentially influencing AD-related pathology via the microbiota-gut-brain axis 15. Nevertheless, direct research examining the anti-AD effects of *Monascus* pigments through this axis remains limited. Compared with monacolin K, *Monascus* pigments appear to have close relevance to anti-inflammation and antioxidant pathways, and complement the lipid-centered actions of monacolin K within the multi-component framework of RYR 10, 14.

### γ-Aminobutyric acid

GABA is a non-protein amino acid, water-soluble, and an inhibitory neurotransmitter widely present in the mammalian central nervous system and peripheral tissues 16, 17. When ingested exogenously, GABA can reduce blood pressure, influence neuroinflammation-related and gut microbiota-related pathways 18, 19. Endogenously produced GABA in the brain is involved in the regulation of cerebral circulation, supports neuronal health and survival, and plays a crucial role in synaptic plasticity underlying learning and memory 17. GABA in RYR may contribute to AD-related neuroprotection through several potential mechanisms, including suppression of neuroinflammation and attenuation of neuronal injury 19, modulation of impaired GABAergic signaling and excitatory/inhibitory imbalance 20, regulation of gut-brain axis communication 21, and improvement of AD-related metabolic risk factors such as hypertension and glucose dysregulation 22, 23.

## Potential Preventive and Therapeutic Effects of the Main Active Substances in RYR on AD

### Cholesterol-Dependent Pathway of Monacolin K in RYR

Studies have demonstrated a correlation between AD and hyperlipidemia 24-26. Initial investigations by Sparks et al. (1994) and Kivipelto et al. (2001) suggested hyperlipidemia as a significant risk factor for AD 24, 25. Subsequent research on apolipoprotein E (ApoE) has further substantiated the link between hypercholesterolemia and AD. Individuals carrying an ApoE-ε4 allele exhibit a threefold increased likelihood of developing AD compared to those without this allele 27, 28. The underlying pathogenic mechanism may involve the APOE-ε4 variant's role in degrading the myelin sheath of oligodendrocytes, which is intricately associated with apolipoprotein's function in lipid transport and metabolism 26. Consequently, maintaining normal blood lipid levels may represent a viable strategy for the prevention and treatment of AD.

The cholesterol-dependent pathway involves the reduction of Aβ peptide levels in the brain by monacolin K from RYR, attributed to its hypolipidemic effect, thereby lowering the risk of AD 29. Buxbaum et al. (2001) conducted cell culture experiments using lipid-depleted serum with or without active lovastatin metabolites 30. They found that lovastatin treatment significantly reduced intracellular Aβ peptide levels under cholesterol-depleted conditions 30. Furthermore, in human subgroups with elevated low-density lipoprotein cholesterol, the application of lovastatin controlled-release formulation or a placebo revealed a decrease in serum Aβ peptide content among hypercholesterolemic patients 31. A cohort study utilizing a longitudinal registry demonstrated that AD patients taking lovastatin exhibited higher Mini-Mental State Examination scores after 3 years compared to non-lovastatin users, with a more pronounced effect observed in patients with hyperlipidemia 32.

Evidence from *in vivo* and *in vitro* studies as well as clinical studies has suggested that the cholesterol-dependent mechanism of monacolin K has positive anti-AD effects in inhibiting Aβ peptide deposition and improving cognition 29-33. This cholesterol-dependent mechanism likely involves lipid rafts, which serve as platforms for processing amyloid precursor protein (APP) into Aβ40 and Aβ42 and thus critically influence Aβ40 and Aβ42 formation 33. Area-Gomez and Schon (2024) proposed the "C99-lipid raft-cholesterol" axis to explain how cholesterol dysregulation promotes Aβ40 and Aβ42 production 33. C99 is a 99-aa membrane-bound C-terminal fragment, generated after APP is cleaved by BACE1 and it is an intracellular lipid regulatory substance that regulates lipid raft formation 33. According to this model, increased cholesterol may facilitate APP endocytosis and intracellular trafficking, leading to C99 increase and the recruitment of cholesterol and other lipids to form lipid raft-like microdomains at mitochondria-associated endoplasmic reticulum membranes. These lipid rafts provide sites for γ-secretase-mediated processing of C99 into Aβ40 and Aβ42 33. Therefore, systemic lipid imbalances may increase Aβ40 and Aβ42 production and alter the Aβ42/Aβ40 ratio by disrupting the C99-lipid raft-cholesterol axis, thereby linking lipid dysregulation to AD pathogenesis 33. These observations support the view that lipid dysregulation contributes importantly to AD pathogenesis and provide a rationale for further exploring lipid-targeted interventions, which aligns with the cholesterol-dependent mechanism of lovastatin in RYR.

### Non-Cholesterol-Dependent Pathways of Monacolin K in RYR

Beyond its cholesterol-dependent mechanism related to AD pathology, monacolin K may also act through a cholesterol-independent pathway that mediates anti-inflammatory and neuroprotective effects 34-36. In an animal study of cerebral malaria, researchers compared chloroquine alone to chloroquine plus lovastatin and found that the lovastatin group showed reduced pro-inflammatory cytokines and oxidative stress markers, along with reversal of cognitive deficits and blood-brain barrier disruption attributable to neuroinflammation 34. These results indicate a pronounced anti-neuroinflammatory and neuroprotective effect. In an experimental rat model involving cognitive impairment, statins increased the anti-inflammatory cytokine IL-10 and reduced neuroinflammation probably via activation of the transforming growth factor-β (TGF-β)/Smad family member 3 (Smad3) pathway 35. Statins also lowered hippocampal levels of chitinase-3-like protein 1 and curtailed excessive glial-cell activation, which may help suppress neuroinflammation in the brains of AD patients 35. The principal mechanism by which lovastatin inhibits neuroinflammation is suppression of excessive glial activation, as evidenced by its ability to reduce glial release of proinflammatory factors such as TNF-α, IL-1β, and IL-6 36.

### Effects of Monacolin K on Aβ peptide and Tau Proteins

Excessive Aβ peptide deposition and phosphorylation of tau proteins are primary pathological hallmarks of AD and a key therapeutic target. Some studies indicate that Aβ peptide deposition and tau aggregation act synergistically in AD pathogenesis, implying that effective prevention and treatment should target both Aβ peptide and tau pathologies simultaneously 37. Lovastatin may influence AD-related Aβ peptide metabolism through lipid-lowering and cholesterol-dependent mechanisms 29, 33, and it may also directly inhibit Aβ40 and Aβ42 formation and promote their clearance 38, 39. The accumulation of Aβ peptides in the brain depends primarily on the balance between Aβ peptide clearance and APP processing 38. Tamboli et al. (2010) reported that lovastatin promotes extracellular Aβ peptide degradation by stimulating exosome-associated insulin-degrading enzyme (IDE) secretion from microglia 40. In a rat experiment, lovastatin significantly reduced brain Aβ42, but this clearance effect on Aβ42 disappeared after IDE gene knockout, indicating that lovastatin accelerates Aβ42 clearance via IDE 38. Lovastatin also alters APP processing-related pathways to lower Aβ peptide levels 38, 39. Endocytosis is a key step in converting APP to Aβ, and one study found that lovastatin inhibits APP endocytosis and downregulates endocytic factors, thereby reducing Aβ42 and Aβ40 formation 39. Xu et al. (2021) experimentally showed that lovastatin down-regulates β-amyloid precursor protein-β-site APP cleaving enzyme 1 (BACE1), thereby inhibiting APP processing to Aβ1-42 38. Hyperphosphorylation of tau is another key therapeutic target in AD, and tau phosphorylation is governed by kinases and phosphatases; their overactivation induces tau hyperphosphorylation. An *in vitro* study found that lovastatin inhibits protein phosphatase 2A, cyclin-dependent kinase 5, and cyclin-dependent kinase 2, thereby reducing excessive tau phosphorylation 41. Because Aβ peptide accumulation interacts with tau pathology during AD progression, lovastatin's concurrent modulation of both pathways may offer an advantage over agents that target only one pathological target. The three anti-AD pathways and mechanisms of the above monacolin K pathways are presented in the diagram (Figure 2).

### Potential Preventive and Therapeutic Effects of *Monascus* pigments on AD

Direct evidence linking *Monascus* pigments to the modulation of Aβ peptide deposition or tau pathology remains limited. Nevertheless, *Monascus* pigments may influence AD-related risk pathways by regulating neuroinflammation, oxidative stress, and hyperglycemia-associated metabolic dysfunction 12, 42. The chronic inflammatory response observed in AD patients can lead to the excessive activation of glial cells, resulting in an overproduction of inflammatory mediators 43. This process exacerbates the accumulation of Aβ peptide and neurofibrillary tangles, ultimately causing neuronal damage and perpetuating a vicious cycle. These findings underscore the significance of neuroinflammation as a key mechanism in the pathogenesis of AD 43.

The anti-inflammatory effects of *Monascus* pigments have been substantiated by previous studies, suggesting their potential role in inhibiting the pathogenesis of chronic neuroinflammation involved in AD processes 44, 45. Lin et al. (2023) reported that *Monascus* pigments can suppress inflammation induced by Aβ40 in mouse neuronal cells, exhibiting significant inhibitory effects on tumor necrosis factor α (TNF-α) and interleukin-6 (IL-6) 12. Notably, the pigment's inhibitory effects on inflammatory factors in the brains of mice injected with Aβ40 in the hippocampus are pronounced 12. Additional research has revealed the mechanisms through which *Monascus* pigments inhibit inflammation. A rat *in vivo* experiment demonstrated that *Monascus* pigments can activate pathways associated with peroxisome proliferator-activated receptor γ (PPARγ), leading to the inhibition of proinflammatory mediators such as tumor necrosis factor-α (TNF-α) and interleukin-6 (IL-6) produced by monocytes 46. Additionally, they reduce inflammatory responses by inhibiting the activities of c-Jun N-terminal kinase (JNK), extracellular signal-regulated kinase (ERK), and Mitogen-Activated Protein Kinase p38 13.

In AD-affected brains, redox imbalance leads to excessive reactive oxygen species (ROS) and reactive nitrogen species (RNS), which may damage neuronal cell membranes, mitochondrial membranes, and proteins, thereby contributing to neuronal dysfunction and degeneration 47. The antioxidant properties of *Monascus* pigments may mitigate oxidative stress and neuroinflammation in the brains of AD patients. An *in vitro* study showed that a rubropunctatin derivative of *Monascus* pigments suppressed Aβ1-42-induced oxidative stress, cell death, and mitochondrial membrane damage in Neuro-2A cells 48, demonstrating a marked neuroprotective effect of *Monascus* pigments *in vitro*. The antioxidant activity of these pigments likely acts synergistically with their anti-inflammatory effects, thereby helping to attenuate late-stage AD-associated neuroinflammation and neuronal injury. *Monascus* pigments and monacolin K differ in their primary modes of action against AD-related pathologies: *Monascus* pigments may create a more favorable brain milieu for inhibiting Aβ peptide and tau pathology by reducing inflammation and oxidative stress 10. In contrast, monacolin K may act more directly through the cholesterol-dependent pathway and Aβ peptide/tau-related mechanisms, as discussed above 29, 37-41.

*Monascus* pigments may provide adjunctive prevention and therapy for AD induced by diabetes or for AD patients with comorbid diabetes. Recent studies indicate that diabetes is a major risk factor for AD, increasing AD risk by approximately 1.52-1.92-fold 49, 50. Evidence suggests that diabetes promotes AD through hyperglycemia-mediated dysregulation of PICALM and mTORC1, which disrupts endosomal function, enhances APP endocytosis, and impairs APP clearance in endosomes, leading to Aβ peptide accumulation in the brain 51. *Monascus* pigments are currently considered one of the principal hypoglycemic components of RYR 52. In particular, ankaflavin and monascin are the main anti-type 2 diabetes metabolites in RYR; they lower blood glucose by reversibly inhibiting α-glucosidase and, compared with acarbose, cause fewer adverse effects 42.

The hypolipidemic properties of *Monascus* pigments have been increasingly recognized. In a controlled study using a hyperlipidemic mouse model, the *Monascus* pigments-treated group exhibited superior lipid-lowering effects compared with the group treated with lovastatin alone 53. This superiority is attributed to *Monascus* pigments' ability not only to reduce lipid accumulation via downregulation of phospholipase expression, but also to modulate lipid metabolic pathways, bile acid biosynthesis pathways, and bile secretion/metabolic pathways that are not influenced by lovastatin 53. The lipid-lowering action of *Monascus* pigments can potentiate the effect of monacolin K; the two agents may engage in complex pharmacokinetic synergism. Consequently, in most experiments, composite RYR extracts demonstrate greater hypolipidemic efficacy than equivalent doses of purified lovastatin 54. Some studies propose that the mechanism by which *Monascus* pigments lower lipids involves activation of PPAR-α and AMP-activated protein kinase (AMPK)-mediated responses, which promote fatty acid β-oxidation, prevent hepatic fatty acid accumulation, increase high-density lipoprotein levels, and decrease total cholesterol, triglycerides, and low-density lipoprotein content 55. The presence of *Monascus* pigments enhances the cholesterol-dependent pathway through which monacolin K exerts its anti-AD effects, and this synergy increases the potential of RYR preparations for the prevention and treatment of AD.

### Potential Preventive and Therapeutic Effects of GABA in AD

Regarding the neuroprotective effects of GABA in AD-related pathologies, studies demonstrate that oral or dietary GABA may suppress and alleviate the neuroinflammatory component of AD-related pathology 19, 56. Although exogenous GABA is generally considered to have limited ability to cross the blood-brain barrier, some studies indicate that the GABA transporter GAT-3, which is highly expressed in astrocytes of the aged brain, can import peripherally derived GABA into the CNS. Once in the brain, GABA can downregulate histone deacetylases 2 and 3 (HDAC2/3) via the CP-CEBPα-miR-34a pathway, thereby reducing HDAC2/3-mediated promotion of proinflammatory cytokine expression and the resulting neuroinflammation and neuronal damage in AD patients 19. Studies suggest that patients with AD exhibit impaired GABA synthesis and receptor function, alongside an excessive release of glutamate and glutamatergic overactivation, which may disrupt the excitatory/inhibitory balance in AD-related neural networks 57. An *in vivo* study in rats demonstrated that chronic oral GABA supplementation increased hippocampal GABA levels and influenced certain memory-related behaviors 58. In addition, indirect human neurophysiological evidence suggests a dietary intervention rich in GABA can modulate excitatory/inhibitory balance 20. Therefore, dietary GABA in RYR may theoretically help modulate AD-related GABAergic dysfunction, but its ability to restore excitatory/inhibitory balance in patients with AD remains to be confirmed. Furthermore, orally administered GABA can influence the nervous system via the gut-brain axis 56. AD-related gut microbiota dysbiosis may alter microbial GABA production by affecting GABA-producing taxa such as Bacteroides and Lactobacillus, thereby influencing GABAergic signaling along the gut-microbiome-brain axis 59. Recent reviews suggest that oral or dietary GABA may influence cognition- and memory-related functions, with evidence from dementia mouse models and elderly populations 21, 56. This potential cognitive effect may be partly linked to GABA-mediated modulation of the gut-microbiome-brain axis, although the precise mechanisms and direct clinical efficacy in patients with AD remain unclear 21, 56. Therefore, the oral intake of GABA from RYR may reach the brain through specific transporters and act via the gut-brain axis, potentially contributing to anti-neuroinflammatory effects, modulation of GABAergic dysfunction, improvement of cognition- and memory-related neural function, and thereby increasing the plausibility of RYR's anti-AD activity.

From the perspective of GABA alleviating chronic metabolic disorders, studies have shown that hypertension is one of the risk factors for AD; long-term hypertension damages the cerebral blood supply system, particularly the circle of Willis, ultimately promoting Aβ peptide deposition and tau protein tangles that precipitate AD 60. GABA exerts antihypertensive effects: in an experiment validating GABA's antihypertensive action, extracts from a high-GABA *Monascus* strain M9011 were administered orally to hypertensive mice. The results indicated that the GABA-rich extract not only completely prevented fructose-induced hypertension but also effectively reversed established fructose-induced hypertension 22. Other studies have demonstrated that GABA promotes pancreatic β-cell proliferation and insulin secretion, thereby exerting anti-diabetic effects; this action can act synergistically with *Monascus* pigments from RYR to lower blood glucose 23, thereby potentiating RYR-associated glucose-lowering pathways relevant to AD prevention.

Monacolin K, *Monascus* pigments, and GABA in RYR exert a multitarget, synergistic effect against Alzheimer's disease (Figure 3), providing a theoretical basis for the potential role of RYR in the prevention and treatment of AD.

## Research on the Prevention and Treatment of AD with RYR Decoction Pieces

### Potential Preventive and Therapeutic Effects of RYR Decoction Pieces on AD via Modulation of the Gut Microbiota

Studies have demonstrated that gut microbiota dysbiosis has been increasingly associated with AD pathogenesis 61. The gut microbiome contributes to neurodevelopment, neurotransmission, and the maintenance of brain homeostasis through microbiota-gut-brain axis pathways 62. Furthermore, it appears to modulate the bidirectional communication between the gastrointestinal tract and the central nervous system in both health and disease. The gut microbiota communicates bidirectionally with the central nervous system through neural pathways including both intrinsic and extrinsic components, endocrine, and immune pathways; this complex signaling network is referred to as the “microbiota-gut-brain” axis 62. Dysbiosis of the gut microbiota, acting through this axis, has been associated with Aβ peptide deposition, tau protein phosphorylation, neuroinflammation, metabolic dysfunction, and oxidative stress 61. *Monascus*-fermented RYR exerts modulatory effects on the gut microbiota. A study indicated that fermented RYR modulates gut microbial composition: lovastatin, *Monascus* pigments, red yeast polysaccharides, and GABA present in SWM008-fermented RYR can increase the abundance of beneficial taxa such as Duncaniella and Akkermansia while suppressing pathogenic bacteria 63. Concurrently, these compounds enhance production of beneficial microbial metabolites, including vitamin K, nucleotide metabolites, and amino acid-derived biomolecules, which may help mitigate dysbiosis-driven induction of AD 63. Exopolysaccharides secreted by *Monascus* during growth serve as significant components with potential prebiotic effects, especially those induced by genistein stimulation (exopolysaccharides, G-EMP). Research indicated that G-EMP can reach the colon, where it is selectively utilized by the gut microbiota 64. This utilization helps preserve the intestinal barrier and inhibit inflammatory pathways, promoting the proliferation of beneficial bacteria, suppressing potentially harmful microbes, and increasing advantageous metabolites such as short-chain fatty acids 64. The lipid-lowering effects of RYR can modulate lipid metabolic dysregulation in the intestines of high-fat diet-fed rats. Analysis of fecal microbiota from these rats revealed that RYR altered gut microbial composition and ameliorated gut dysbiosis induced by hyperlipidemia 65. By modulating the gut microbiota, RYR may effectively reduce neuroinflammation and rectify brain dysfunction via the gut-microbiota-brain axis, thereby exerting anti-Alzheimer's disease effects.

### Experimental Study on the Anti-AD and Neuroprotective Effects of Red Yeast Rice Decoction Pieces

Current research on RYR concerning its anti-Alzheimer's disease and neuroprotective properties remains limited, primarily focusing on* in vitro* and *in vivo* studies. One *in vivo* investigation revealed that RYR offers neuroprotective effects in a mouse model of Parkinson's disease through antioxidant and anti-neuroinflammatory mechanisms, suggesting its potential for the treatment or prevention of nervous system disorders 66. Conditions of zinc deficiency in the brain, which have been associated with AD, contribute to age-related memory decline and hippocampal pathology 67. In an *in vivo* study involving zinc-deficient rats, the administration of RYR significantly enhanced antioxidant enzyme activity and neuronal function, while also preserving cortical and hippocampal integrity, irrespective of zinc supplementation status 68. Although these findings do not directly demonstrate anti-AD effects of RYR, they suggest that the antioxidative and anti-neuroinflammatory activities of RYR-related metabolites may warrant further investigation in AD-related contexts, particularly in relation to oxidative stress, neuroinflammation, memory impairment, and hippocampal injury 66, 68. Lee et al. (2008) demonstrated *in vitro* that an extract of RYR fermented by *Monascus* purpureus NTU568 can alleviate Aβ40-induced toxicity in PC12 cells through its antioxidant and anti-inflammatory properties 69. The study included a comparator group treated with an equivalent dose of lovastatin alone and found that the RYR extract resulted in a more pronounced reduction of Aβ40 toxicity, along with enhanced antioxidant and anti-inflammatory effects, which the authors attributed to synergistic interactions among multiple components 69. In an *in vivo* study involving rats, an ethanol extract of RYR was administered to a hyperlipidemic AD rat model that received an intracerebroventricular injection of Aβ40; the RYR extract significantly reversed memory deficits. Mechanistic analyses indicated that these effects may be mediated by the inhibition of β-secretase activity, leading to decreased Aβ40 accumulation, and by the promotion of amyloid precursor protein processing in the hippocampus toward the production of the neuroprotective sAPPα 70, 71. A recent experimental study in the amyloid precursor protein (APP) transgenic J20 mouse model of AD showed that RYR treatment reduced hippocampal Aβ peptide levels, enhanced brain cholinergic activity, attenuated neuroinflammation, and reduced release of proinflammatory cytokines 45. The study proposed that the anti-AD mechanism of RYR may involve activation of PPAR-γ; among the potential downstream effects of PPAR-γ activation are inhibition of BACE1-mediated Aβ40 and Aβ42 production, reduction of tau hyperphosphorylation, and modulation of neuroinflammation 45. These findings indicate the neuroprotective potential of RYR *in vivo* and in animal models, highlighting its multi-target anti-AD properties. However, clinical research remains limited.

## Discussion

### Potential advantages of RYR in AD-related modulation

The pathogenesis of AD is intricate and multifactorial, often occurring alongside metabolic disorders such as hyperlipidemia, hypertension, diabetes, and metabolic syndrome. These chronic metabolic conditions and AD interact synergistically, mutually reinforcing each other. Therefore, prevention and therapeutic strategies for AD should adopt a multifaceted approach targeting various angles and mechanisms. Current synthetic targeted pharmaceuticals encounter significant limitations, including restricted therapeutic efficacy and challenges in managing adverse reactions. A comprehensive therapeutic strategy that simultaneously modulates Aβ peptide production and clearance, inhibits excessive tau phosphorylation, alleviates neuroinflammation, improves metabolic disorders, and regulates gut microbiota homeostasis may hold greater promise than single-target drugs. Metabolites found in RYR—such as monacolin K, *Monascus* pigments, and GABA, may exhibit multi-effect, multi-target synergistic actions 45,69, providing antihyperlipidemic, antidiabetic, antihypertensive, antioxidant, anti-neuroinflammatory, anti-Aβ peptide deposition, tau phosphorylation-related, and gut microbiota-modulating properties 22, 42, 45, 65, 69. However, current research on the synergistic effects of various RYR metabolites in AD-related pathologies remains limited, and further research is needed. These attributes indicate significant potential for RYR in the prevention and treatment of complex disorders such as AD. From the perspective of long-term disease progression, the onset of AD is protracted and insidious during its early stages. Once established, AD proves challenging to treat. Consequently, RYR may warrant further evaluation as a preventive measure and an early intervention for AD, potentially mitigating its progression associated with various metabolic disorders and facilitating timely intervention in the initial stages.

### Safety standards and citrinin control

Although RYR is a natural fermented product with multi-component and multi-target characteristics, it should not be regarded as inherently free of safety concerns. Because monacolin K is chemically identical or closely related to lovastatin, monacolin K-containing RYR preparations may produce statin-like adverse effects, including hepatotoxicity, myopathy, and, rarely, rhabdomyolysis 72. In addition, citrinin, a nephrotoxic mycotoxin that can be produced during *Monascus* fermentation, represents an important quality and safety concern for RYR preparations 73. Citrinin contamination is particularly relevant when considering long-term preventive or adjunctive use of RYR in AD-related populations, who are often older and may have comorbid renal, hepatic, or metabolic disorders. Therefore, any future development of RYR-based interventions for AD should prioritize standardized production, quantitative monitoring of monacolin K and citrinin, and the use of well-characterized citrinin-free or low-citrinin-producing *Monascus* strains 74.

### Limitations and perspectives

Currently, medicines derived from RYR have been utilized for lipid reduction with favorable outcomes. However, their application in the prevention and treatment of AD remains largely theoretical. While research has demonstrated that RYR and several of its constituents possess anti-AD properties, clinical studies are limited. Aside from lovastatin, which has been subjected to clinical trials for AD treatment 75, RYR preparations and other constituents have not undergone clinical evaluation. Consequently, advancing RYR toward clinical application for AD prevention and therapy presents several challenges. Future initiatives may need to initiate long-term epidemiological studies in high-risk populations or conduct trials involving combination drug regimens. Investigating the anti-AD activities of the various secondary metabolites in RYR, along with their potential synergistic interactions, could clarify the multi-target cooperative advantages of RYR for AD prevention and treatment, suggesting a promising avenue in the anti-AD domain.

## Figures and Tables

**Figure 1:**
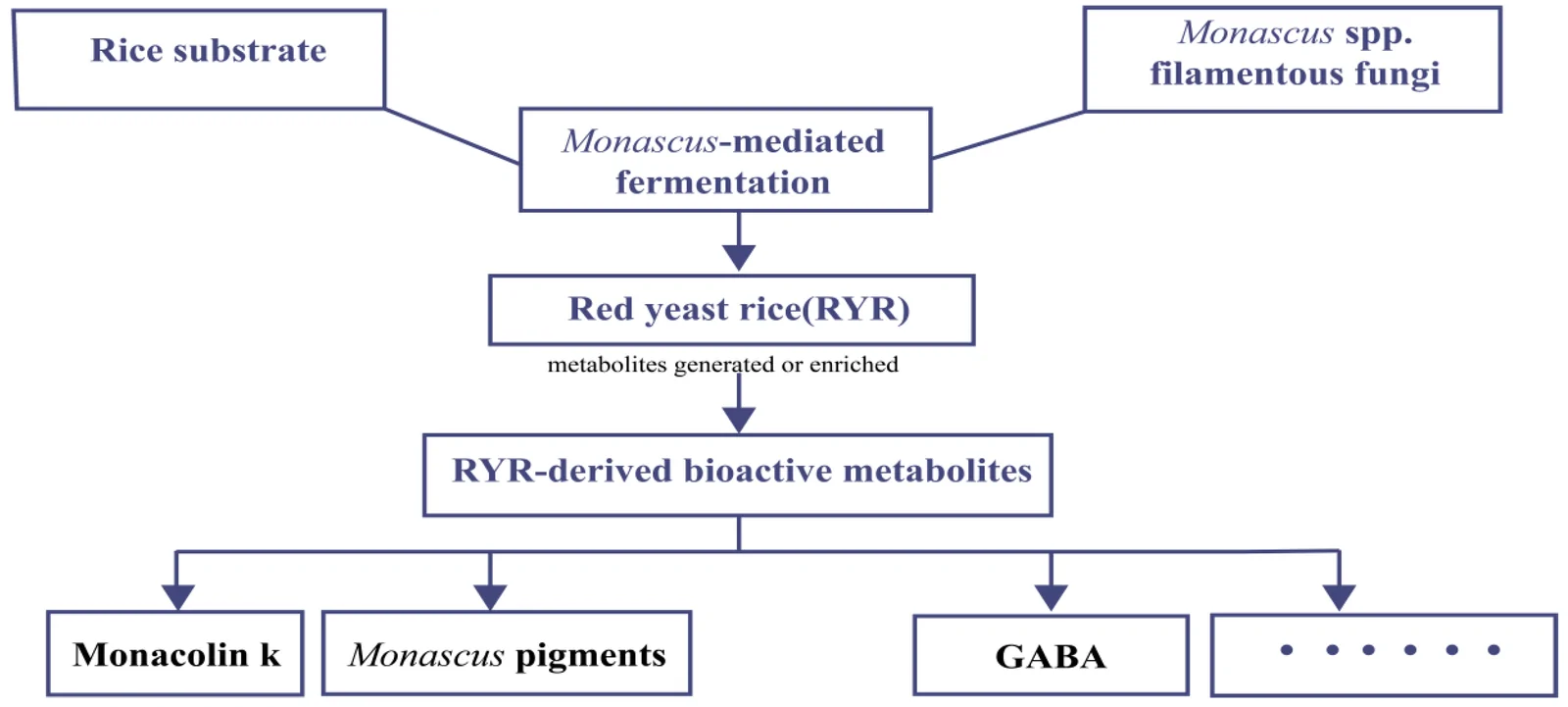
Schematic overview of the relationship among RYR, Monascus, and the major bioactive metabolites generated or enriched during Monascus-mediated fermentation.

**Figure 2:**
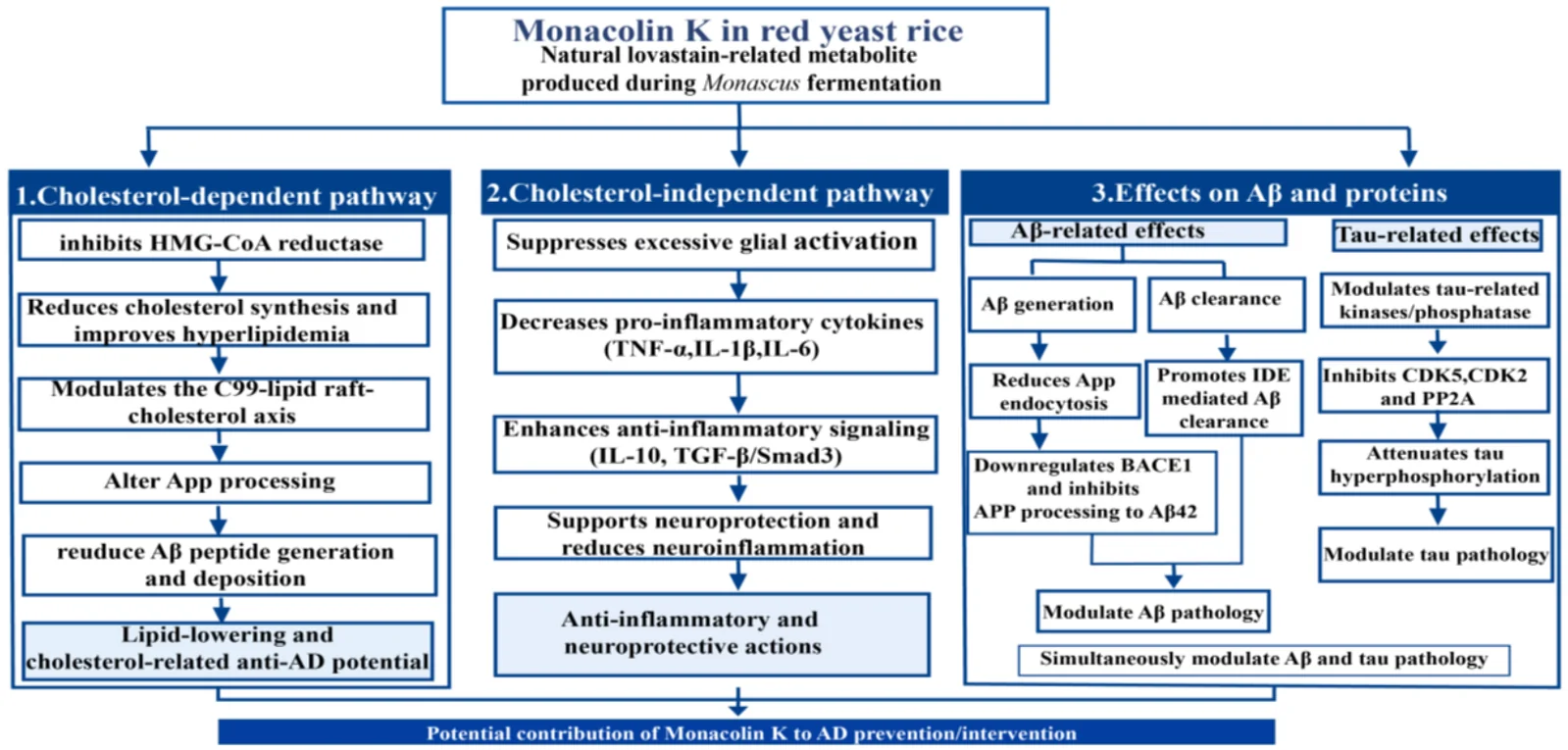
Three pathways and main mechanisms by which monacolin K in RYR may modulate AD-related pathology.

**Figure 3:**
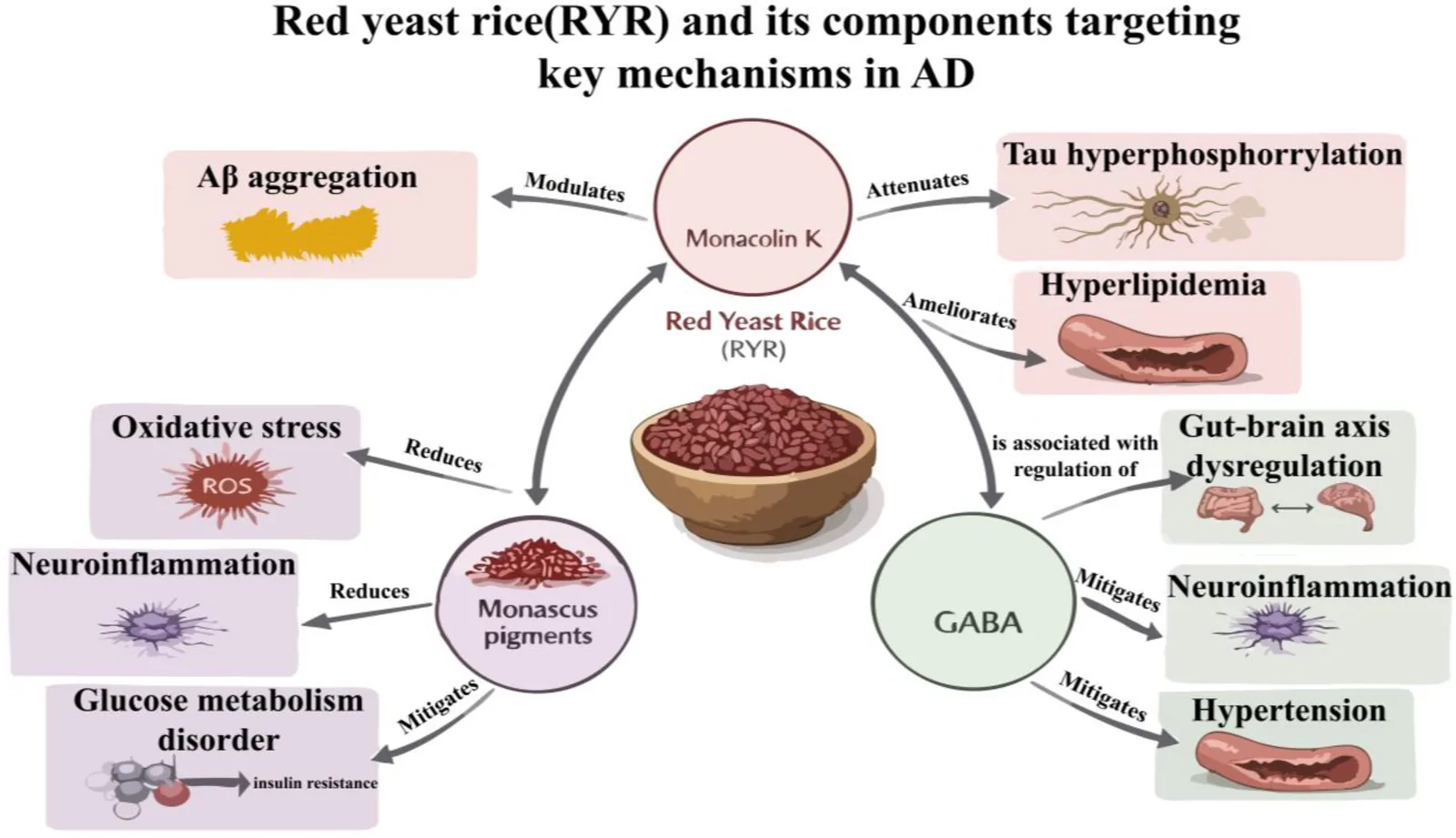
Mechanisms by which the active constituents of RYR exert therapeutic effects on AD.
